# Benefits of dual-release hydrocortisone treatment on central adiposity and health-related quality of life in secondary adrenal insufficiency

**DOI:** 10.1007/s40618-022-01940-1

**Published:** 2022-10-17

**Authors:** V. Gasco, J. Giannelli, L. Campioni, E. Arvat, E. Ghigo, S. Grottoli, M. Maccario, R. Giordano

**Affiliations:** 1 Division of Endocrinology, Diabetes and Metabolism, Department of Medical Sciences, University of Turin, Turin, Italy; 2 Division of Oncological Endocrinology, Department of Medical Sciences, University of Turin, Turin, Italy; 3 Division of Endocrinology, Diabetes and Metabolism, Department of Medical Sciences and Department of Clinical and Biological Sciences, University of Turin, Corso Dogliotti 14, 10126 Turin, Italy

**Keywords:** Dual-release hydrocortisone, Adiposity, Metabolism, HRQoL, Hypothalamic–pituitary disease

## Abstract

**Purpose:**

Patients with secondary adrenal insufficiency (SAI) have an increased morbidity and an impaired health-related quality of life (HRQoL), which seems to primarily depend on the sub-optimal replacement of hypoadrenalism with standard glucocorticoid (GC) therapy, and on the inadequate correction of other associated pituitary deficiencies. A dual-release hydrocortisone (DR-HC) formulation has shown to exert positive effects on morbidity and HRQoL, mainly in patients with primary adrenal insufficiency. We assessed the variations of anthropometric and metabolic parameters and HRQoL in patients with SAI after switching from cortisone acetate (CA) or hydrocortisone (HC) to DR-HC.

**Methods:**

Twenty-one patients (17 M, 4 F) treated with CA (*n* = 16; 25 mg/day twice a day) or HC (*n* = 5; 20 mg/day three times a day), were evaluated for waist circumference, BMI, fasting glucose, HbA1c, insulin, HOMA-IR index, serum lipids, electrolytes, blood pressure and HRQoL at baseline, at 3, 6 and 12 months after switching from CA/HC to DR-HC.

**Results:**

The study showed a significant reduction of waist circumference and BMI (*p* = 0.04, for both), after 3 and 6months of DR-HC treatment, respectively. No significant changes were observed for fasting glucose, insulin, HOMA-IR index, HbA1c, total cholesterol, triglycerides, LDL cholesterol, electrolytes, and blood pressure. However, HDL cholesterol significantly decreased (*p* = 0.003). An improvement of AddiQoL total score was observed during DR-HC treatment (*p* = 0.01), mainly for the category “emotions”. No predictors resulted for these changes.

**Conclusion:**

DR-HC treatment provides some benefits in patients with SAI, reducing central adiposity and improving HRQoL; however, worsening of HDL cholesterol is observed during treatment with DR-HC.

## Introduction

Secondary adrenal insufficiency (SAI) is a rare but life-threatening endocrine disorder, characterized by inadequate cortisol secretion mainly due to hypothalamus–pituitary damage [1–3].

Patients with SAI need life-long glucocorticoid (GC) replacement therapy, which is necessary for the maintenance of vascular tone [1–3]. Despite GC replacement therapy, patients with SAI present increased morbidity and impaired health-related quality of life (HRQoL) compared to the general population [1–3]. These abnormalities could be secondary to both supra-physiological GC doses used in the past (> 20 mg/day hydrocortisone, HC) [4], or to a more physiological GC doses used nowadays (≤ 20 mg/day HC), but unable to replicate the endogenous cortisol circadian rhythm [5].

Therefore, novel pharmaceutical GC have been studied to improve morbidity and HRQoL in patients with adrenal insufficiency. Among them, a once-daily dual-release HC formulation (DR-HC, Plenadren^®^) has been developed to maintain cortisol levels in a more physiological range than conventional GC treatment [6].

Studies performed on patients with primary adrenal insufficiency (PAI) treated with DR-HC have already established an improvement on some anthropometric (i.e., body weight reduction), metabolic (i.e., HbA1c reduction), cardiovascular (i.e., blood pressure reduction) parameters, and HRQoL, compared to standard GC replacement therapy [7–10]. To date, studies on the use of HC-DR in SAI were few (including retrospective data) [11, 12] or small [9, 13–15]. These studies generally reported a reduction in BMI and, in some cases, in waist circumference, but they are less concordant with an improvement in glucose and lipid metabolism as well as with HRQoL [9, 11–16]. Furthermore, data were biased by the fact that in some studies, the switch from conventional GC therapy to DR-HC involved the transition from clearly supra-physiological to physiological doses [9, 11, 14, 15], and therefore the improvements observed might have been related to the reduction of the dose rather than to the drug formulation.

Based on this background, we designed a clinical prospective study aimed at evaluating the effect of switching from physiological conventional GC treatment (cortisone acetate, CA, or HC) to DR-HC replacement treatment on anthropometric and metabolic parameters and on HRQoL in patients with SAI, after 3, 6 and 12 months of follow-up. Moreover, we sought to identify characteristics that might be useful in selecting patients who could benefit more than others from the transition from conventional GC treatment to DR-HC therapy.

## Subjects and methods

### Subjects

We performed an open-labeled, non-randomized, prospective, monocentric study.

Eligible patients were males and females aged ≥ 18 years, with a diagnosis of secondary adrenal insufficiency [basal serum cortisol lower than 3.0 µg/dl (82.7 nmol/l) or standard corticotrophin-stimulated cortisol below 18 µg/dl (500 nmol/l), accordingly to the Endocrine Society Guidelines [3]], who were treated with conventional CA or HC replacement therapy, without any adjustment in hormone replacement for at least 6 months before study entry.

Exclusion criteria included patients aged < 18 years; pregnancy; patients with acute medical or surgical illness; concomitant severe cerebral, cardiovascular, respiratory, renal, gastrointestinal (i.e., hepatobiliary/pancreatic diseases, gastrointestinal emptying or motility disturbances) disorders; patients treated with GC for purposes other than adrenal insufficiency; patients treated with any drugs which interfere with corticosteroid metabolism including oral estrogen-containing drug due to the effect on corticosteroid-binding globulin and thus total cortisol concentrations; and patients with known Type 1 or Type 2 diabetes mellitus requiring insulin therapy.

All patients gave their written informed consent to participate in the study, which had been approved by the Ethical Committee of the University of Turin, in agreement with the principles of the Declaration of Helsinki.

After screening for eligibility and informed consent, patients were switched from their usual GC regimen to a once-daily dose of DR-HC 20 mg/day administered orally in the morning at fasting, and evaluated after 3, 6 and 12 months. All patients were instructed to add a rescue dose of CA (12.5 or 25 mg) or HC (5 or 10 mg) according to severity of stress and symptoms during an intercurrent illness or stress. In case of inability to take oral therapy or in case of reduced gastrointestinal absorption (vomiting or diarrhea), patients were instructed to take HC (25 or 50 mg trice a day according to severity of the clinical picture) intramuscularly.

### Evaluation of clinical parameters and health-related quality of life

Body mass index (BMI), waist circumference and blood pressure were measured using standard methods at baseline and after 3, 6 and 12 months of treatment with DR-HC.

HRQoL was evaluated by a 30-item questionnaire (AddiQoL), purposely developed and validated for patients with Addison’s disease [17, 18]. The AddiQoL identifies four sub-dimensions: fatigue (8 items), emotions (8), symptoms (9) and miscellaneous (sleep, sexuality and impact of intercurrent disease, 5). The algebraic sum of the various items’ scores was calculated: a higher score indicated a better HRQoL.

### Biochemical evaluation

Blood samples were collected from all patients at fasting, between 8:00 and 9:00 AM, before and 120 min after drug administration. Blood fasting glucose, insulin, HbA1c, total and high-density lipoprotein (HDL) cholesterol, triglycerides, electrolytes, cortisol, and IGF-I were directly measured, while low-density lipoprotein (LDL) cholesterol was calculated by Friedewald equation.

Serum glucose levels (mg/dl; 1 mmol/l = 18 mg/dl) were measured by enzymatic method based on hexokinase reaction, according to isotope dilution/mass spectrometry (ID/MS) standards.

Serum total cholesterol (mg/dl; 1 mmol/l = 38.6 mg/dl), and triglycerides (mg/dl; 1 mmol/l = 88.5 mg/dl) levels were measured by enzymatic colorimetric assay, according to ID/MS standards.

Serum HDL cholesterol levels (mg/dl; 1 mmol/l = 38.6 mg/dl) were measured by enzymatic colorimetric homogeneous test (immuno-separation followed by enzymatic cholesterol assay), standardized according to the US national reference system for cholesterol measurement, Cholesterol Reference Method Laboratory Network – CRMLN.

All the above-mentioned analyses were performed on Roche/Hitachi Cobas automated platforms (Roche Diagnostics), being the coefficient of variations < 1.9% for both within-run and between-run evaluations.

HbA1c levels (mmol/mol) were measured by gold standard ion-exchange high-performance liquid chromatography (HPLC, Tosoh Bioscience, Inc., San Francisco, CA, USA), with the inter-and intra-assay coefficients of variation lower than 2%.

Serum insulin levels (µUI/ml; 1 pmol/l = 0.14 µUI/ml) were measured by automated chemiluminescence immunoassay on Immulite 2000 (Siemens Healthcare Diagnostics, Deerfield, USA).

Basal insulin sensitivity was evaluated by the homeostasis model assessment of insulin resistance (HOMA-IR) index [19].

Serum Na and K were measured using ion-selective electrode system (Cobas ISE module), fully automated on routine clinical biochemistry analysers.

Serum cortisol levels (µg/dl; 1 nmol/l = 0.036 µg/dl) were measured by automated immunoassay on Cobas e601 instrument (Roche Diagnostics GmbH, Mannheim, Germany), based on a competitive electro-chemiluminescence immunoassay, with an analytical sensitivity of 0.018 µg/dl, and intra- and inter-assay precision ranging from 3.0 to 5.7%, and from 2.4 to 6.2%, respectively.

Serum IGF-I levels (μg/l; 1 nmol/l = 7.65 μg/l) were measured in duplicate by RIA method (SM-C-RIA-CT, DIAsource ImmunoAssays, Belgium) after acid–ethanol extraction to avoid interference by binding proteins. The sensitivity of the method was 0.25 μg/l. The inter- and intra-assay CV were 6.8–14.9% and 4.5–7.0%, respectively. IGF-I levels are expressed as a standard deviation score (SDS) of the mean normal value. The SDS for each subject was calculated in accordance with the published normality data on a population of 547 healthy Italian subjects [20].

### Statistical analysis

Statistical analysis was performed using STATA 17 (StataCorp, College Station, Texas, USA). The results are expressed as median and interquartile range (IQR) for continuous variables and percent value for categorical variables. Changes in clinical, biochemical and HRQoL values were studied by non-parametric Friedman and Kendall and Wilcoxon matched-pairs signed-rank test, where appropriate. Possible predictors of significant changes in clinical variables and HRQoL values (expressed as absolute difference between median value at 12 months and baseline) during DR-HC treatment were investigated by Kruskal–Wallis rank test and Fisher’s exact test for univariate models and by multivariate logistic regression analysis.

## Results

**Fig. 1:**
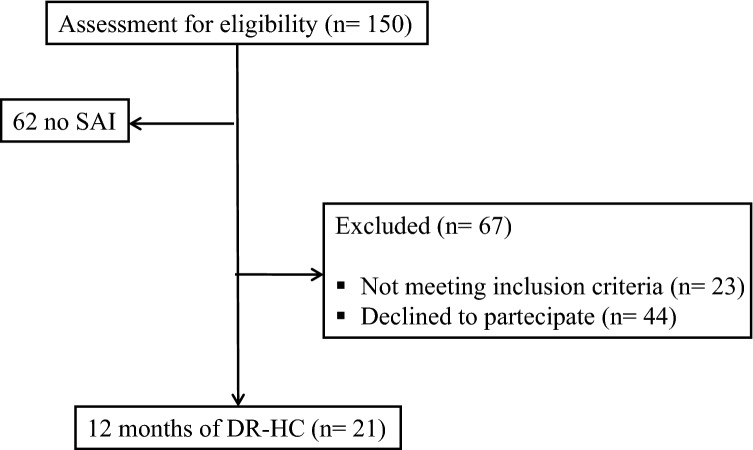
Consort diagram of the study population

From January 2016 to March 2016, the first 150 consecutive patients referred to the Neuroendocrinology Clinic in our Centre were screened for eligibility. A consort flow diagram of the study population is outlined in Fig. 1. Twenty-one patients [17 M and 4 F; median age 44 (11.5) years] entered the study.

All patients presented at least one other pituitary deficiency, which had already been set under appropriate hormonal replacement therapy for a period of at least 6 months before the enrollment, except for 8 patients (cases 2, 3, 5, 9, 12, 14, 18, 19) with severe acquired GH deficiency (GHD), who have not been treated with GH because of concomitant-specific contraindicating conditions (e.g., concomitant comorbidities, poor patient compliance). Case number 7 had active acromegaly at baseline and subsequently showed good disease control during GH antagonist therapy.

At baseline, patients were under CA (25 mg/day, BID: *n* = 16) or HC (20 mg/day, TID: *n* = 5) treatment, that had been administered at the same dose for at least 6 months before.

At baseline, 2 patients had normal body weight, 12 were overweight (BMI 25–30 kg/m^2^) and 7 were obese (BMI > 30 kg/m^2^). Five patients had arterial hypertension, 3 had type 2 diabetes mellitus and 5 had dyslipidaemia.

The main patient clinical features at baseline are reported in Table 1.

**Table 1:** Clinical data of patients with SAI

Case	Gender	Age (years)	BMI (kg/m^2^)	Etiology	Associated pituitary deficiencies under treatment	Disease duration (years)
1	M	42	22.7	PNS (CRA)	APH, DI	25
2	M	44	29.2	PNS (macro-NS)	APH	2
3	M	56	23.9	TBI	APH, DI	40
4	F	42	27.7	PNS (macro-NS)	APH	5
5	M	72	34.7	Empty sella	APH	2
6	M	37	26.0	PNS (macro-NS)	APH	3
7	M	43	26.6	PNS (micro GH +)	HT	5
8	F	42	30.6	Hypophysitis	HT, HG	1
9	M	53	27.4	PNS + RT (germinoma)	APH, DI	25
10	M	61	26.0	PNS (macro-NS)	APH	26
11	M	57	31.2	Empty sella	APH	16
12	M	40	33.6	Congenital hypoplasia	APH	40
13	M	48	26.6	Empty sella	APH	14
14	M	38	32.7	PNS (CRA)	APH, DI	13
15	F	42	28.0	PNS (CRA)	APH, DI	4
16	F	50	26.8	PNS (meningioma)	HT	4
17	M	36	25.9	PNS (macro-NS)	APH	2
18	M	53	29.1	Congenital hypoplasia	APH	20
19	M	45	31.2	PNS (CRA)	APH, DI	2
20	M	30	27.2	PNS (CRA)	APH, DI	22
21	M	51	31.7	PNS (macro-NS)	APH	23

*M* male, *F* female, *BMI* Body Mass Index, *PNS* post neurosurgery, *RT* radio therapy, *macro-NS* not-secreting macro-adenoma, *macro* macro-adenoma, *micro* micro-adenoma, *CRA* cranio-pharyngioma, *GH* + GH-secreting adenoma, *PRL* + PRL-secreting adenoma, *TBI* traumatic brain injury, *APH* anterior pan-hypopituitarism, *HT* hypothyroidism, *HG* hypogonadism, *DI* diabetes insipidus, *GHD* GH deficiency

Clinical and biochemical data are reported in Table 2.

**Table 2:** Clinical and biochemical parameters (median and 25°–75° quartiles) in patients with SAI during conventional GC treatment (HC or CA), and after 3 (3 M), 6 (6 M) and 12 (12 M) months of dual-release hydrocortisone (DR-HC)

	HC or CA	DR-HC 3 M	DR-HC 6 M	DR-HC 12 M	*p*
BMI (kg/m^2^)	27.7 (26.6–31.2)	27.5 (25.8–30.1)	27.3 (26.0–29.5)	27.2 (25.6–30.6)	**0.04**
Waist circumference (cm)	95 (93–106)	94 (90–100)	92 (88–97)	93 (88–99)	**0.005**
PAS (mmHg)	122 (115–130)	120 (110–130)	120 (110–130)	125 (100–130)	0.55
PAD (mmHg)	80 (75–82)	80 (70–82)	80 (70–80)	80 (75–80)	0.86
Na + (mmol/l)	142 (140–143)	142 (141–144)	142 (140–143)	143 (141–145)	0.12
K + (mmol/l)	4.1 (3.9–4.3)	4.3 (4.0–4.4)	4.1 (4.0–4.4)	4.2 (4.0–4.4)	0.67
Glucose (mg/dl)	78 (72–86)	82 (74–89)	86 (78–90)	82 (78–89)	0.16
Insulin (µU/ml)	7.2 (4.0–8.8)	8.3 (4.9–15.9)	7.9 (3.5–10.1)	6.5 (5.0–8.2)	0.89
HOMA-IR	1.4 (0.9–2.4)	1.6 (0.9–3.1)	1.8 (0.9–2.2)	1.5 (1.0–3.3)	0.65
HbA1c (mmol/mol)	36 (33–39)	38 (36–40)	38 (34–41)	38 (34–40)	0.17
Total cholesterol (mg/dl)	186 (165–194)	184 (158–202)	189 (157–206)	186 (153–217)	0.82
Triglycerides (mg/dl)	120 (95–148)	129 (105–179)	129 (99–174)	116 (97–193)	0.78
HDL cholesterol (mg/dl)	51 (45–55)	41 (36–50)	43 (34–51)	41 (35–48)	**0.003**
LDL cholesterol (mg/dl)	105.6 (94–118.4)	103 (92.2–121)	110.6 (97.2–132.8)	108.4 (89.4–132.6)	0.83
Cortisol 120 min after drug ingestion (µg/dl)	17.0 (13.1–18.0)	16.4 (13.8–17.3)	16.4 (13.7–18.0)	15.2 (12.8–17.3)	0.72
IGF-I SDS	−0.23 (−1.25 to 0.29)	−0.56 (−0.48 to 1.18)	−0.70 (−1.11 to 0.30)	−0.51 (−1.26 to 0.02)	0.57

Bold values indicate stastical significativity of p value (< 0.05)

Fasting glucose, insulin, HOMA-IR index, HbA1c, total serum cholesterol, triglycerides, LDL cholesterol and electrolyte levels remained stable during treatment with DR-HC. Neither systolic nor diastolic blood pressure levels significantly changed. No significant changes were detected in cortisol levels measured 2 h after drug administration, and in IGF-I SDS during DR-HC treatment.

**Fig. 2:**
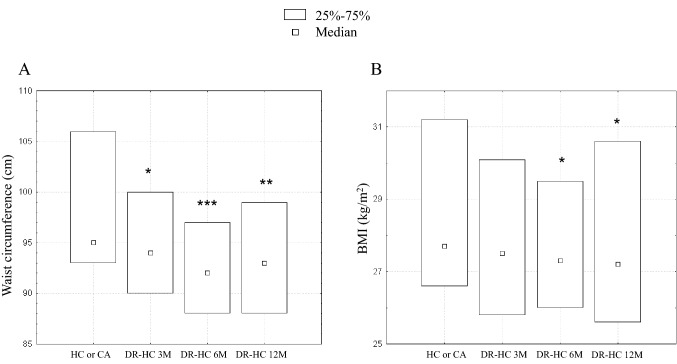
Waist circumference (cm) (**A**) and BMI (kg/m.^2^) (**B**) (median, 25° and 75° centiles) during conventional GC treatment (HC or CA) and after 3 (3 M), 6 (6 M) and 12 months (12 M) of dual-release hydrocortisone (DR-HC), in patients with SAI. *p < 0.05 vs baseline; **p < 0.01 vs baseline; ***p < 0.005

A progressive reduction was observed in waist circumference and BMI values, already significant after 3 months of treatment [94 (10) vs 95 (13) cm, *p* = 0.04] and 6 months [27.3 (3.5) vs 27.7 (4.6) kg/m^2^, *p* = 0.04], respectively (Fig. 2). Similarly, HDL cholesterol significantly decreased (*p* = 0.003); this variation occurred already after 3 months of treatment [41 (14) vs 51 (11) mg/dl, *p* = 0.002].

Patients with lower IGF-I SDS values at baseline (*p* = 0.006) and those with untreated GHD (*p* = 0.03) were more prone to waist reduction (Table 3).

**Table 3:** Univariate analysis of possible predictors of significant changes (expressed as absolute difference between median value at 12 months and baseline) of BMI, waist circumference, and HDL cholesterol in patients with SAI after switch from conventional GC treatment (HC or CA) to dual-release hydrocortisone (DR-HC)

	BMI decrease ≥ 0.5 kg/m^2^ Y vs N	*p*	Waist decrease ≥ 2 cm Y vs N	*p*	HDL cholesterol decrease ≥ 10 mg/dl Y vs N	*p*
CA (%)	78.6 vs 71.4	0.56	66.7 vs 88.9	0.26	100 vs 68.7	0.22
HC (%)	21.4 vs 28.6		33.3 vs 11.1		0 vs 31.3	
F (%)	14.3 vs 28.6	0.41	33.3 vs 0	0.08	20 vs 18.8	0.70
M (%)	85.7 vs 71.4		66.7 vs 100		80 vs 81.2	
Age (years)	46.5 (40–56) vs 42 (42–53)	0.43	43 (41–51.5) vs 48 (42–53)	0.67	51 (42–53) vs 43.5 (40–51.5)	0.51
Neurosurgery (%)	64.3 vs 71.4	0.57	58.3 vs 77.8	0.32	60 vs 25	0.18
Other (%)	35.7 vs 28.6		41.7 vs 22.2		40 vs 75	
Disease duration (years)	8.5 (2–23) vs 20 (5–25)	0.60	4 (2–16.5) vs 22 (14–25)	0.06	23 (16–25) vs 5 (2.5–21)	0.28
Total pituitary deficit (%)	92.7 vs 71.4	0.25	83.3 vs 89.9	0.61	80 vs 87.5	0.60
Others (%)	7.1 vs 28.6		16.7 vs 11.1		20 vs 12.5	
GHRT or no-GHD (%)	57.1 vs 71.4	0.44	41.7 vs 89.9	**0.03**	60 vs 62.5	0.66
GHD (%)	42.9 vs 28.6		58.3 vs 11.1		40 vs 37.5	
Baseline BMI (kg/m^2^)	28.6 (26–31.7) vs 27.4 (26.6–29.1)	0.41	29.1 (27.3–31.9) vs 26.6 (26–27.4)	0.12	31.2 (30.6–31.7) vs 27 (26–29.1)	**0.04**
BMI < 30 kg/m^2^ (%)	57.1 vs 85.7	0.21	58.3 vs 77.8	0.32	20 vs 81.3	**0.02**
BMI ≥ 30 kg/m^2^ (%)	42.9 vs 14.3		41.7 vs 22.2		80 vs 18.7	
Waist circumference (cm)	95.5 (88–106) vs 95 (93–97)	0.91	96.5 (91–106) vs 93 (93–97)	0.41	97 (97–106) vs 94.5 (88–101.5)	0.16
Baseline IGF-I SDS < −1 (%)	42.8 vs 28.6	0.83	66.7 vs 0	**0.006**	20 vs 43.8	0.38
Baseline IGF-I SDS > −1 and < + 1 (%)	42.9 vs 57.1		25 vs 77.8		80 vs 37.5	
Baseline IGF-I SDS > + 1 (%)	14.3 vs 14.3		8.3 vs 22.2		0 vs 18.7	
Baseline cortisol 120 min after drug ingestion (µg/dl)	16 (13.1–18) vs 18 (12.6–21.3)	0.50	17.8 (14–18.1) vs 15.9 (12.6–18)	0.34	18 (18–21.3) vs 16 (12.8–18)	0.22

Bold values indicate stastical significativity of p value (< 0.05)

*CA* cortisone acetate, *HC* hydrocortisone, *F* female, *M* male, *GHRT* growth hormone deficiency on rhGH treatment, *GHD* growth hormone deficiency, *BMI* Body Mass Index, *SDS* standard deviation score, *Y* yes, *N* no

Patients with higher BMI [both as absolute value (*p* = 0.04) and as categorical expression of obesity vs non-obesity (*p* = 0.02)] were more prone to HDL cholesterol reduction (Table 3).

No predictors were confirmed at multivariate analysis both for waist circumference and HDL cholesterol.

No significant predictors of BMI reduction were even highlighted at univariate analysis (Table 3).

AddiQoL data are reported in Table 4.

**Table 4:** AddiQoL scores (median and 25°–75° quartiles) in patients with SAI during conventional GC treatment (HC or CA), and after 3 (3 M), 6 (6 M) and 12 (12 M) months of dual-release hydrocortisone (DR-HC)

AddiQoL	Fatigue	Emotions^§^	Symptoms	Miscellaneous	Total^§^
HC or CA	21 (18.5–23.5)	23 (19.5–24)	27 (25.5–29)	14 (12–15.5)	86 (79–82)
DR-HC 3 M	22 (20–24)	23.5 (21.5–25.5)*	28 (25.5–30)	14 (12.5–15.5)	90 (78–93.5)*
DR-HC 6 M	23 (21–24)	24 (22.5–24.5)	28 (25.5–29.5)	15 (13–15.5)	87.5 (82.5–90.5)**
DR-HC 12 M	22 (19.5–23.5)	24 (23–24.5)***	26 (24–28)	14.5 (13–15.5)	88.5 (82.5–93)^§§^

Fatigue: items 1–5, 23, 26 and 27; Emotions: items 11–15, 24, 25 and 30; Symptoms: items 6, 9 and 16–22; Miscellaneous: items 7, 8, 10, 28 and 29

*p = 0.04

**p = 0.03

***p = 0.01

^§^*p* = 0.01 for trend

^§§^*p* = 0.003 vs baseline

The total AddiQoL score increased during DR-HC treatment (*p* = 0.01), with a significant improvement vs baseline at all time points [3 months vs baseline: 90 (15.5) vs 86 (3), *p* = 0.04; 6 months vs baseline: 87.5 (8) vs 86 (3), *p* = 0.03; 12 months vs baseline: 88.5 (10.5) vs 86 (3), *p* = 0.003]. Mean score for emotions sub-dimension significantly increased at 3 months [23.5 (4) vs 23 (4.5), *p* = 0.04] as well as at 12 months [24 (1.5) vs 23 (4.5), *p* = 0.01]; no significant changes were detected in mean scores for remaining sub-dimensions (fatigue, symptoms and miscellaneous) at any time (Table 4).

Younger patients (*p* = 0.05) and those with a lower total AddiQoL at baseline (*p* = 0.02) were more prone to total AddiQoL increase (Table 5). Patients with a lower total AddiQoL at baseline (*p* = 0.04) and a lower AddiQoL score in emotions sub-dimension (*p* = 0.04) were more prone to AddiQoL increase in emotions sub-dimension (Table 5). The data were not confirmed at the multivariate analysis both for total AddiQoL and for emotions sub-dimension.

**Table 5:** Univariate analysis of possible predictors of significant changes (expressed as absolute difference between median value at 12 months and baseline) of AddiQoL in patients with SAI after switch from conventional GC treatment (HC or CA) to dual-release hydrocortisone (DR-HC)

	AddiQoL TOT increase ≥ + 2.5 Y vs N	*p*	AddiQoL emotions increase ≥ + 1 Y vs N	*p*
CA (%)	66.7 vs 85.7	0.39	63.6 vs 100	0.18
HC (%)	33.3 vs 14.3		36.4 vs 0	
F (%)	33.3 vs 0	0.15	27.3 vs 0	0.30
M (%)	66.7 vs 100		72.7 vs 100	
Age (years)	42 (37–42) vs 48 (43–53)	**0.05**	42 (37–50) vs 48(43–53)	0.16
Neurosurgery (%)	77.8 vs 57.1	0.37	81.8 vs 40	0.14
Other (%)	22.2 vs 42.9		18.2 vs 60	
Disease duration (years)	5 (3–22) vs 16 (5–25)	0.31	5 (2–23) vs 16 (14–25)	0.17
Total pituitary deficit (%)	53.9 vs 46.1	0.60	69.2 vs 30.7	0.71
Other (%)	66.7 vs 33.3		66.7 vs 33.3	
GHRT or no-GHD (%)	77.8 vs 57.1	0.37	72.7 vs 60	0.52
GHD (%)	22.2 vs 42.9		27.3 vs 40	
Baseline BMI (kg/m^2^)	26.8 (25.9–27.7) vs 29.2 (26.6–31.7)	0.12	27.2 (25.9–30.6) vs 27.4 (26.6–31.2)	0.46
BMI < 30 kg/m^2^ (%)	77.8 vs 57.2	0.37	72.7 vs 60	0.52
BMI ≥ 30 kg/m^2^ (%)	22.2 vs 42.8		27.3 vs 40	
Waist circumference (cm)	94 (88–97) vs 95 (93–106)	0.43	95 (88–97) vs 95 (93–97)	0.99
Baseline IGF-I SDS < −1 (%)	44.4 vs 28.6	0.82	45.5 vs 20	0.33
Baseline IGF-I SDS > −1 and < + 1 (%)	44.4 vs 42.8		45.5 vs 40	
Baseline IGF-I SDS > + 1 (%)	11.2 vs 28.6		9.0 vs 40	
Baseline cortisol 120 min after drug ingestion (µg/dl)	14 (12.6–18) vs 17.6 (13.1–21.3)	0.46	15.9 (12.6–18) vs 17 (13.1–21.3)	0.78
Baseline AddiQoL TOT	80 (74–85) vs 89 (87–95)	**0.02**	81 (74–88) vs 89 (88–95)	**0.04**
Baseline AddiQoL emotions	20 (19–23) vs 24 (23–24)	0.06	21 (19–23) vs 24 (24–24)	**0.04**

Bold values indicate stastical significativity of p value (< 0.05)

*CA* cortisone acetate, *HC* hydrocortisone, *F* female, *M* male, *GHRT* growth hormone deficiency on rhGH treatment, *GHD* growth hormone deficiency, *BMI* Body Mass Index, *SDS* standard deviation score, *Y* yes, *N* no

The most commonly reported side effect was fatigue (2/21 patients, 9.5%), which transiently occurred in the first month of DR-HC treatment. During the study period, only 2 out of 21 patients needed rescue oral therapy (in both cases for no more than three consecutive days) and no patient needed parenteral HC therapy. No severe adverse event was reported.

Finally, no significant dose adjustment for thyroid, gonadal and GH replacement treatment as well as for the treatment of hypertension, dyslipidaemia or diabetes was required.

## Discussion

Our study demonstrated a significant reduction of central adiposity and BMI as well as an improvement of HRQoL associated with DR-HC replacement therapy in patients with secondary adrenal insufficiency previously treated with conventional GC; however, a worsening of HDL cholesterol was observed during treatment with DR-HC. Strong predictors of the anthropometric, biochemical and HRQoL changes induced by DR-HC could not be identified, however, based on data from the univariate analysis, we were able to identify some characteristics that might be useful in selecting patients who could benefit more than others from the transition from conventional GC treatment to DR-HC therapy. DR-HC also appeared well tolerated and safe, since only two patients reported transient fatigue, while no major side effect was reported.

SAI patients need life-long GC replacement therapy to replace cortisol deficiency [1–3]. However, conventional GC therapy has been associated with increased morbidity, mainly obesity and glycometabolic alterations [3, 21, 22] and impaired HRQoL [23–27], in comparison with the general population. This may be due to inability of conventional GC treatment to adequately replicate the physiological cortisol circadian rhythm, resulting in peaks followed by rapid decline of cortisol levels, and high late-afternoon/evening cortisol levels [28–31]. Moreover, the coexistence of other pituitary deficiencies, inadequately or not entirely replaced, can contribute to the increased cardiovascular morbidity and impaired HRQoL in SAI patients [3, 32].

Some previous studies have explored different HC formulations, trying to identify the best doses and patterns of replacement treatment in patients with hypocortisolism.

A DR-HC formulation was developed to maintain cortisol levels in a more physiological range, as clearly demonstrated in a phase I study [6]. DR-HC consists in an immediate releasing coat and in an extended-release core, providing a peak of GC during the morning followed by a gradual decrease during the day. Cortisol exposure in terms of area under the curve with DR-HC is 20% lower to that obtained with conventional GC therapy [7, 9, 10].

To date, most of the data relating to the effects of switching from conventional GC therapy to DR-HC treatment mainly concerned patients with PAI; these studies showed a significant reduction in body weight, blood pressure, and improvement of glucose metabolism and HRQoL in PAI patients [7, 9, 10].

However, data concerning the role of DR-HC in SAI are scanty, with most studies including retrospective data [11, 12] or few patients [9, 13–15]. These studies generally report a reduction in BMI and, in some cases, in waist circumference, but they are less consistent on glucose, lipid metabolism and HRQoL improvement [9, 11–16].

Furthermore, the data are biased by the fact that sometimes the studied populations include both PAI and SAI patients, not always differentiating the results on the nature of the adrenal insufficiency [9, 11, 13]; moreover, in some studies, the switch from conventional GC therapy to DR-HC involves the transition from clearly supra-physiological to physiological doses [9, 11, 14, 15], and therefore it cannot be excluded that the improvements observed are related to the reduction of the dose rather than to the formulation type.

Finally, to date, there are no data in SAI patients regarding any predictors of a greater benefit in switching from conventional GC to DR-HC therapy.

In agreement with previous studies, we found a significant reduction in waist circumference and BMI values already 3 and 6 months, respectively, after switching from conventional GC to DR-HC treatment. Patients who had the lower IGF-I SDS levels at baseline (possible expression of an untreated or inadequately replaced GHD) and untreated GHD patients would seem to be those who could benefit most in terms of waist circumference reduction during DR-HC therapy.

Even if the absence of a body composition evaluation could represent a minor study limitation, as it would have better characterized differences in fat distribution before and after DR-HC treatment, our results seem to suggest that DR-HC is able to modify central adiposity mostly in GHD patients when GHD is under or no-replaced, while the improvement induced by DR-HC does not appear to occur in no-GHD patients or in patients with correctly substituted GHD.

It remains to be clarified if the lack of replacement of concomitant severe GH deficiency can affect and, in case, at which extent, the potential benefit of replacement with DR-HC on other anthropometric parameters.

According to our results, DR-HC treatment did not significantly affect metabolic parameters, including fasting glucose, insulin, HbA1c levels as well as HOMA-IR index, suggesting that influence of DR-HC on glucose metabolism in SAI is not as strong as found in PAI. These data are in accordance with some [14–16], but not all [9, 11–13] previous studies on DR-HC treatment in SAI. However, it is surprising that despite the observed waist circumference and BMI reduction the HOMA index did not change. This could be due to the fact that although the BMI reduction was significant, it did not result in a variation in the distribution of lean, overweight and obese subjects in our study group. At baseline, there were 2 lean, 12 overweight and 7 obese patients; 12 months after the therapeutic switch from conventional therapy to DR-HC, there were 3 lean, 12 overweight and 6 obese patients. A similar argument can be made about waist circumference: although the observed reduction of this parameter was significant, this did not, however, lead to a variation in the classes of central obesity in the study group. At baseline, there were 8 (6 M and 2 F) patients who had a waist circumference > 102 cm if male and > 88 cm if female; at 12 months from the therapeutic switch the patients were 7 (5 M and 2 F).

It remains to be clarified whether, with prolonged treatment and observation time, the reduction in BMI and waist circumference will be confirmed; if this was the case, it is also likely to expect a reduction in the HOMA index.

Moreover, no significant changes in total and LDL cholesterol as well as in triglycerides levels were detected, confirming what was found in previous studies performed in patients treated with DR-HC [7, 9, 12, 14]. However, some former studies [15, 16] also reported an increase in triglycerides and a reduction in HDL cholesterol [15, present study] levels in SAI patients during DR-HC treatment which deserves further investigation. Glucocorticoids have complex, still not fully explained effects on glucose and lipid metabolism, including direct and indirect actions on gluconeogenesis, glycolysis, lipolysis, free fatty acid synthesis and turnover, very-low-density lipoproteins synthesis and fatty storage in liver [33, 34]. It remains debated whether the lack of replacement treatment in some of our GHD patients could have affected the potential benefit of DR-HC on lipid metabolism in SAI, since GC tissue exposure increases in GHD patients not substituted [35–37]. However, beyond intrinsic methodological limitations, it is important to observe that our patients appeared adequately replaced for thyroid and gonadal axis, since endocrine insufficiencies other than GH have demonstrated strong modulating effects on both glucose and lipid parameters.

Concerning the reduction of HDL cholesterol levels during DR-HC therapy, it is not surprising that it occurs more frequently in more obese patients, due to the known inverse correlation between adiposity and HDL cholesterol [38].

Finally, we have confirmed the previously reported improvement in HRQoL associated with DR-HC therapy [7, 9, 10, 15, 39, 40]. Our study group reported an improvement of AddiQoL total score during DR-HC treatment, mainly for the category “emotions”. Inadequate GC replacement therapy is suspected to be a major cause of reduced HRQoL in adrenal insufficiency, although evidence has been lacking [23, 41]. Immediate release HC given in divided dosed BID or TID is associated with considerable variability in cortisol concentrations, with a substantial proportion of patients being undertreated and over-treated during the same 24-h period [42]. DR-HC avoids these multiple daily peaks and troughs in cortisol levels, which may translate into the observed improvement in HRQoL for these patients.

Regarding the improvement in HR-QoL during DR-HC therapy, it is not surprising that it occurs more frequently in younger patients or patients with greater HR-QoL impairment at baseline; in fact, in hypopituitaric and in PAI patients, HR-QoL tends to be better in youngers [18, 43] and to improve more during therapy, especially in patients with more compromised values at baseline [27, 43].

As demonstrated in patients with PAI [7–10], DR-HC appears extremely safe in patients with SAI, since a similar prevalence of minor adverse events was recorded between conventional HC and DR-HC, being transient, rapidly recovering fatigue the most common reported adverse event. We suppose this phenomenon could be related more to an increased awareness of associated signs and symptoms, than to a real change in cortisol regimen, although changes in cortisol exposure time cannot be ruled out.

Our study presents some limitations. First, patients were not blinded, so their expectations on the efficacy of the new drug could have, at least partially, affected results. Second, the study population was relatively small with a clear preponderance of male gender, and this probably compromised the multivariate analysis outcome. Lastly, patients were heterogeneous, especially with regards to replacement of GH deficiency. These last data make further studies necessary to support our observation that patients with untreated GHD seem to benefit most from DR-HC treatment.

Despite these limitations, our study represents a new attempt to evaluate the impact of DR-HC treatment in patients with SAI. Moreover, even if we failed to identify strong predictors of a better response to DR-HC, probably due to the relatively small group of patients, our study highlights the relevance of assessing some baseline anthropometric and hormonal parameters, as well as HRQoL in a cohort of patients with secondary adrenal insufficiency under replacement, for a more proper definition of the impact of the various GC.

In conclusion, our results support the hypothesis that replacement with DR-HC is advantageous in patients with SAI, since it reduces central adiposity and improves HRQoL compared to conventional GC treatment; however, a worsening of HDL cholesterol levels is observed during treatment with DR-HC. Younger patients, those with a lower AddiQoL, BMI, IGF-I SDS values, and those with untreated or not adequately substituted GHD at baseline seem to benefit most from DR-HC treatment. Larger, long-term, and randomized blind case–controlled studies are required to confirm these preliminary findings and identify stronger predictors of outcome.
